# Qatar mental health law: Knowledge and attitude among healthcare workers

**DOI:** 10.5339/qmj.2026.57

**Published:** 2026-08-25

**Authors:** Majid Alabdulla, Shuja Mohd Reagu, Mugtaba Osman, Denise Haddad, Sultan Albrahim, Yousaf Iqbal

**Affiliations:** 1 Mental Health Services, Hamad Medical Corporation (HMC), Doha, Qatar; 2 College of Medicine, Qatar University, Doha, Qatar; 3 Independent Researcher, Manchester, United Kingdom; 4 Naufar Center, Qatar Addiction Treatment and Rehabilitation Centre, Doha, Qatar

**Keywords:** Medicolegal aspects, human rights, legal rights, Middle East, Qatar

## Abstract

**Background:**

Qatar’s mental health law (MHL) is designed to regulate the management of individuals with mental illness and to safeguard their rights and is currently in the process of implementation. Studies from countries with established MHL systems reveal varying attitudes of stakeholders towards this law. This study aimed to assess awareness and attitudes of healthcare workers (HCWs) to proposed MHL provisions in Qatar.

**Method:**

A cross-sectional survey using a specially designed online questionnaire was carried out. In addition to socio-demographic information, responses to questions exploring attitudes to MHL provisions were recorded on a 5-point Likert scale, which were then analysed statistically and thematically. The survey was carried out from 10^th^ October 2021 to 30^th^ November 2021, at the state-run Hamad Medical Corporation (HMC), Qatar.

**Results:**

Over a thousand (*n* = 1006) respondents participated in the survey. The Cronbach’s alpha estimate for the attitudes subsection of the questionnaire was 87% (95% CI: 86% to 88%). This is indicative of a very good internal consistency. The overall mean attitude score was 57.6 points (out of a maximum possible 80 points), indicative of a generally positive attitude (SD = 9.6 points). Most respondents agreed that the law will have a positive impact on patient rights, access to treatment, timely assessment, and management. However, only around a third (35.3%) reported that they had received any training around the law. Thematic analyses highlighted that respondents believed that the law would align local practice with International human rights standards and that the implementation would improve mental healthcare in general and improve access to care. Concerns around a lack of adequate training and a lack of focus on the mental health of healthcare staff were the other main themes.

**Conclusion:**

This is the first and the largest survey of its kind in the region and highlights that there is an overall positive attitude towards this law in Qatar among HCWs. This augurs well for the full implementation and operationalisation of this law.

## 1. INTRODUCTION

Qatar passed a dedicated mental health law (MHL) in 2016 following a rigorous and wide-ranging consultation process.^1–4^ Although Qatar is not the first nation in the region to develop a specialised MHL,^5–7^ Qatar, however, drafted its law informed by the shortcomings and feedback to similar existing laws in the region.^1^

The MHL aims to address key areas in mental health care.^1^ The law consists of 7 chapters comprising 35 articles and offers a legal framework for the assessment and treatment of individuals with mental illness, including provisions for informed consent. The law defines mental disorder and regulates voluntary and involuntary admission to hospital. It emphasises protection of patients’ rights, including dignity, privacy, access to information and the right to appeal against involuntary admission. It also formalises processes for capacity and consent, recognises the role of guardians and mandates the establishment of the Competent Authority to review compulsory admissions and safeguard patient rights. The Competent Authority ensures that compulsory care is justified and that patients have access to an appeals process. It operates independently to ensure fair and lawful treatment decisions. The law mandates patients’ rights to information, privacy, and treatment options. It also defines clear roles for guardians in decision-making.

Strict procedures for compulsory admissions are outlined in the law, requiring a second opinion from an independent psychiatrist. Patients have the right to appeal compulsory admissions, access second opinions, and receive care that respects their dignity. Informed consent is central to the law, with assessments of patient capacity documented. The law also includes provisions for Compulsory Community Care, allowing patients to receive mandated care outside the hospital^8^ while maintaining public safety. It provides a legal framework for recalling patients to the hospital if necessary. The impact of the COVID-19 pandemic and Qatar’s limited number of trained psychiatrists have delayed the full implementation and operationalization of the law.

Qatar has ratified the Convention on the Rights of Persons with Disabilities (CRPD)^9^, which includes a commitment to ensuring the rights of individuals with mental health conditions. The draft Code of Practice of the MHL, the Competent Authority framework, and the extensive training of frontline clinicians on the MHL are aligned with the principles of the CRPD.

Studies from nations with well-established MHL systems have shown that various stakeholders- doctors, patients, caregivers, the criminal justice system, and the general public have diverse perspectives toward the use of involuntary admission under the MHL.^10–13^ The attitudes vary across countries and even among various stakeholders within the same country, with healthcare workers (HCWs) usually having more positive attitudes than other stakeholders, like the criminal justice system, patients, and carers.^14,15^ Positive healthcare attitudes to MHL are probably because mental health workers see the benefits of the law first hand in reducing suffering and securing the rights of patients.^15^ Even within HCWs, the attitudes to mental ill health varied across countries, and in countries where work has been undertaken on lessening stigma associated with mental ill health, there were more progressive attitudes.^16,17^

A recent narrative review described the evolution of mental health and substance use policy and legislation in Qatar and highlighted the limited published literature in this area.^18^ Available research comparing mental health professionals with those not working in the field suggests that effective implementation of MHL requires ongoing training and educational programmes, active engagement of key stakeholders, and a sustained focus on protecting patients’ rights and quality of care.^19,20^

The current study, whilst exploring attitudes to MHL among HCWs, differs from available evidence in that previous data are for attitudes to a fully established MHL system, whereas this study explores these attitudes in a system where the law is yet to be fully operationalised. This study aimed to assess awareness and attitudes of HCWs to proposed MHL provisions in Qatar. It is pertinent to mention that Hamad Medical Corporation (HMC), Qatar, employs HCWs from over 100 countries, some of whom come from countries with established MHL systems, while others do not.^21^ It is hoped that the results from this study will aid effective implementation and stakeholder engagement in Qatar and add to the relatively limited global literature on this subject.

## 2. METHODS

A cross-sectional, convenience sampling method was used to recruit participants for this study. HMC is Qatar’s principal state-run health system, which delivers the majority of healthcare to Qatari residents at highly subsidised rates or free of cost.^21,22^ HMC is the principal provider of in-patient mental health beds and will be the primary health system in Qatar that will use the MHL when fully operationalised. HMC employs over 25000 HCWs, and the survey was sent as an online link using the corporation staff database. Ethical approval for the study was obtained from the HMC’s Institutional Review Board (MRC-01-21-304). Participation in the study was voluntary. The completion of the anonymous survey was taken as implied consent.

A specially designed questionnaire was developed by the research team, comprising physicians with experience in psychiatry, as such a study has not been attempted before in this context. The questionnaire was informed by existing instruments, including the Mental Health Legislation Attitudes Scale, the World Health Organization Assessment Instrument for Mental Health Systems (WHO-AIMS), and prior qualitative studies on MHL implementation.^15,23,24^ It was adapted to reflect the specific provisions and anticipated implementation of Qatar’s MHL.

The questionnaire comprised three subsections: The first collected demographic data and respondents’ own experience of mental health systems in Qatar or anywhere else; the second assessed attitudes towards MHL provisions using a Likert-scale format; and the third allowed respondents to provide free-text responses regarding the implementation and utility of the MHL in Qatar.

The questionnaire underwent face and content validation and was reviewed by subject matter experts in mental health services (MHS). A formal pilot study was not undertaken; however, the questionnaire was reviewed internally for clarity and relevance prior to distribution.

The questionnaire was sent electronically to all of HMC staff on 10^th^ October 2021, and the survey closed on 30^th^ November 2021, by which time over 1000 responses were achieved. The survey was closed after responses had been received from a broad range of HCWs, including those working within and outside mental health services, and from physicians and allied health professionals.

### 2.1. Statistical analysis

Descriptive statistics were used to analyse demographic characteristics; counts for clinical factors, proportions for categorical variables, and mean and standard deviation for numerical variables. Based on their favorability in terms of a positive attitude toward the implementation of the MHL, attitude components were given a 1–5 score per component. The final analysis assigned 4 points for ‘agree’, 5 points for ‘strongly agree’, 1 point for ‘strongly disagree’, and 2 points for ‘disagree’. We awarded 3 points for the ‘undecided’ response. A total attitude score was calculated by adding up the numerical equivalence of the agreement level. We carried out regression analyses for the total attitude score against the background demographic factors simultaneously to evaluate their impact.

The total score was predicted to fall between a minimum of 16 and a maximum of 80 due to the 16 numerical items used. Through the use of Cronbach’s alpha estimation, the internal consistency was evaluated. ANOVA (Analysis of Variance) was used to evaluate the impact of independent variables against dependent variables, such as age, on attitude score. To investigate two-level variables like sex, t-test analysis was used to assess their impact on attitude. Multiple linear regression was performed to investigate the adjusted impact of background factors on the overall attitude score. The free-text question provided at the end of the questionnaire was investigated using a qualitative inductive thematic analysis method. Descriptive and inferential analysis were performed using R Statistical Software 3.6.0 (R Core Team, 2020).

## 3. RESULTS

We received 1006 valid responses from HCWs in the online survey. The Cronbach’s alpha estimate for the 16-item questionnaire was 87% (95% Confidence Interval: 86–88%). The overall mean attitude score for all the responses was 57.6 points (out of 80 points) (SD = 9.6 points), ranging from a minimum of 16 points to a maximum of 80 points. The median score was 58 points.

Table 1 shows the descriptive results for the background demographic characteristics and their individual and unadjusted impact on attitude score. In terms of age distribution, the youngest category was 18–24 years old (*n* = 5, 0.5%) of all participants, who also scored the lowest mean attitude score of 49 points. The modal category was the 25–34 years old (*n* = 393, 39.1%), followed by the 35–44 years old (*n* = 384, 38.2%); their attitude scores were comparable: 57.7 and 57.3 points, respectively. Notably, the highest attitude score was observed among those over 65-years old (63.3 points). However, age category did not significantly affect attitude score at the unadjusted level (*F* = 1.294, *p* = 0.264). Males were (*n* = 344, 34.2%) of all participants, and Qataris were (*n* = 23, 2.3%). However, the impact of sex and nationality on attitudes was non-significant. On the other hand, (*n* = 217, 21.6%) of participants were MHS workers. Their attitude significantly exceeded that of participants with no mental health affiliation (60.4 points vs 56.8 points in terms of mean attitude score, *p* < 0.00001).

Table 2 shows each of the attitude components towards MHL broken down according to the Likert Scale. Only 35.3% agreed that they are well-informed about Qatar’s MHL, compared to 63.6%, who felt either uninformed or unsure. Moreover, 76% believed that Qatar’s MHL will offer protection for the rights of patients admitted to hospital, and 78.4% agreed that Qatar’s MHL will reduce the risk of self-harm among psychiatric patients. About 74.7% agreed that Qatar’s MHL will reduce the risk to others from patients with severe mental health problems. Further, 81.9% of respondents believed that Qatar’s MHL will improve the quality of healthcare offered to patients with mental illness. Around 79.6% of the respondents were content that Qatar’s MHL implementation will lead to timely access to effective treatments for mental illnesses.

Of the respondents, 76.2% believed that Qatar’s MHL implementation will ensure timely access to see a mental health professional (mental health care provider). We found 70.8% agreeing that Qatar’s MHL will reduce stigma towards mental disorders. 76.1% of those who responded believed that patient should access a second independent opinion regarding their diagnosis and treatment. An appeal process for patients against their own detention was seen as a valuable feature of the MHL by 55.3% of those who responded. This number was higher at 62% when asked for the right of appeal by the patients’ carers. 46.9% of the respondents agreed that patients should be subjected to compulsory treatment by their treating team under the MHL.

In terms of awareness and confidence in training, only about a third (35.4%) of the respondents stated that they had received training and were confident that they were ready for the full implementation of the MHL. Finally, just 10% of the respondents believed that the implementation of the MHL would have little to no impact on their clinical practice. Furthermore, upon exploring the impact of nationality on these attitudes, none of these attitudes showed any statistically significant difference in the attitudes of Qatari HCWs and HCWs of immigrant extraction.

Direct comparison between Qatari and non-Qatari HCWs was equivocal in terms of attitudes towards MHL in Qatar. The mean attitude score among Qatari HCWs was 57.8 points (compared to a mean attitude score of 57.6 points among their non-Qatari counterparts). This difference was not statistically significant (*t* = 0.093, *p* = 0.9266).

**Figure 1.**
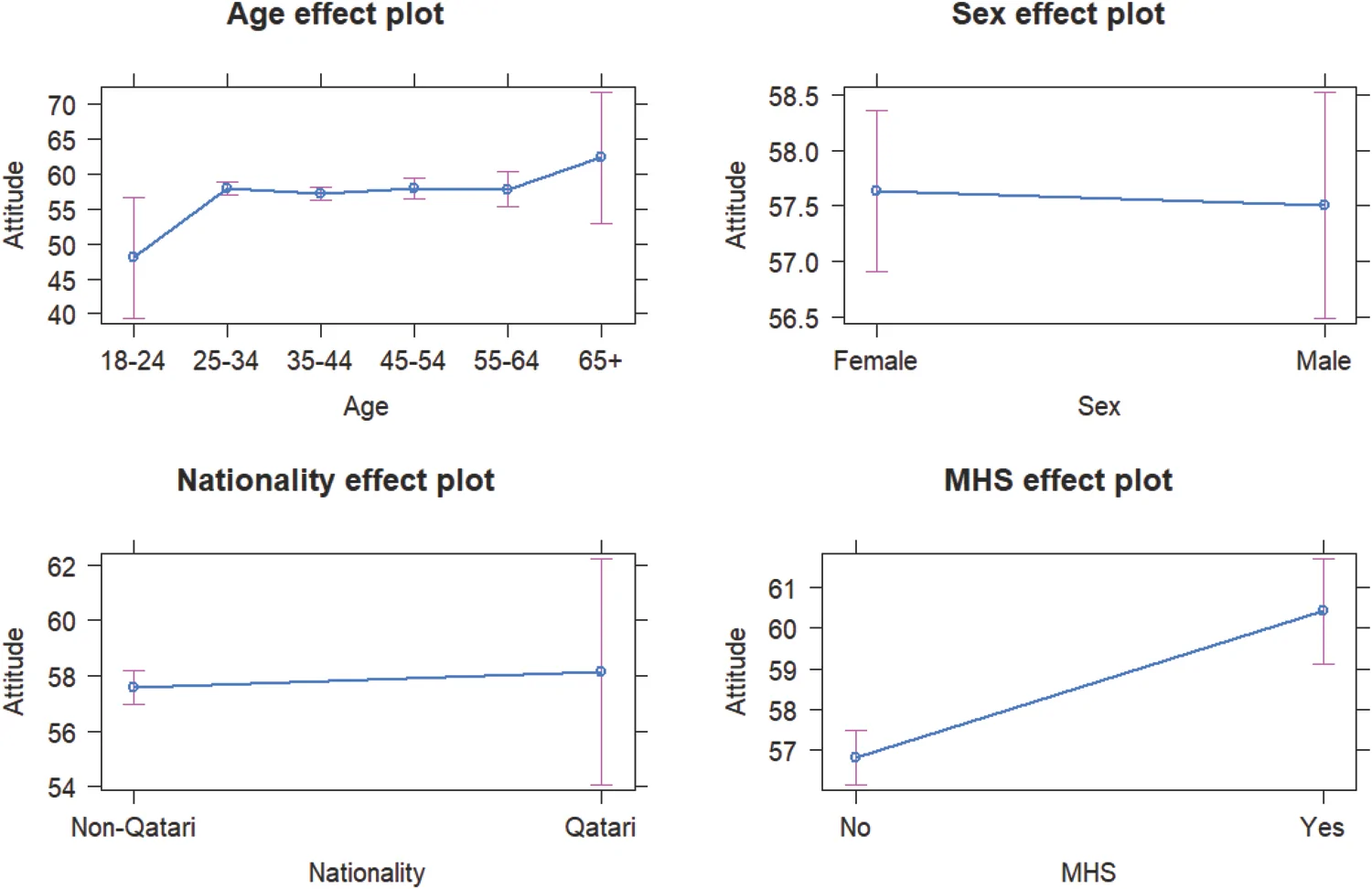
Visual representation of the effects of background factors on attitude towards MHL among study participants.

In terms of the adjusted effect for background factors on attitude score towards MHL in Qatar, as shown in Table 3 and Figure 1, there was a clear positive impact for older age (compared to younger respondents) on attitudes, with the difference most stark between those under 24 years of age compared with those over 65 years. Being affiliated to MHS had a significant impact on attitudes’ score by 3.6 points (95% CI: 2.1 to 5.1, *p* < 0.00001). Furthermore, when we examined nationality effects broken down according to establishment of MHL, we could not identify any difference in terms of attitudes.

### 3.1. Thematic analysis of question 17

This offered an opportunity for participants to share their views using free-text style with regard to the implementation of MHL in Qatar. We identified 6 main themes as follows:

***Theme 1: Training and awareness:*** A vast majority of the responses highlighted the need for increased awareness and widespread training for HCWs before full implementation.

*“MH Staff need enhanced training before implementation of MHL”. “I am new in the hospital and the country, hence why my knowledge is limited. However, following this survey, I will actively look into the subject to familiarize myself with it”.*

***Theme 2: Conform to international human rights standards:*** MHL was seen as ensuring conformity to international human rights standards by many HCWs.

*“Mental health awareness is so poor in Qatar, I’m very hopeful that this law will bring HMC into the present international mental health arena. My personal concern is the small number of mental health professionals available in HMC, but maybe this is already being addressed. “Good job, doing best for good of humankind, proud to work in Qatar and be a part of Hamad General Hospital.”*

***Theme 3: Protection of rights:*** HCWs saw the MHL will ensure protection of individual rights when receiving mental health care.

*“I feel that MHL is very important for patient rights as well as for HCWs”. “It is deemed helpful to both locals and expatriates.”*

***Theme 4: Access to care:*** MHL was seen as improving access to care.

*“Implementation and provide a platform to access the MHL”.*

***Theme 5: Concerns around coercion:*** HCWs raised concerns about the protection from coercion under the MHL.

*“Still, I’m not sure about this: Should a patient, admitted to a hospital under Qatar’s MHL, be able to be given treatment against their will by the psychiatric team? I’m going to re-review the final version to make sure about the correct practice.”*

***Theme 6: Staff poor mental health and burnout:*** Interestingly, HCWs used this survey to highlight the need for focus on the mental health of the staff itself.

*“Please do a survey on the mental health status of the Nurses working in different facilities. We are overworked, understaffed, stressed, and deeply fatigued. Has long working hours (12Hrs) but inadequate rest days, causing us fatigue and severe anxiety”.*

## 4. DISCUSSION

This is the first such study that explores the attitudes of HCWs to MHL in the Middle Eastern and North African (MENA) region, and that has been done in a yet-to-be-established MHL system that the authors are aware of. A key finding of this study is that, despite relatively limited awareness of the detailed provisions of the MHL, the majority of HCWs viewed it as a positive development and as an instrument likely to protect patient rights, improve access to care, and reduce stigma. This apparent discrepancy may partly reflect the influence of broader trust in health systems, as policies perceived as fair, transparent, and aligned with the public interest are more likely to gain stakeholder support despite limited detailed understanding; similarly, a recent study found broadly positive professional attitudes towards mental health legislation, while also highlighting important gaps in awareness, implementation, and training.^25^ It is likely that respondents were expressing attitudes towards the perceived purpose and principles of the law, such as protection of patient rights and improved access to care, rather than its specific operational details.^26^ Furthermore, low awareness may reflect limited existing training, as there was one day of training arranged for selected MHS staff. A recent study found that caregivers generally recognised the importance of mental health legislation and supported further seminars and workshops to improve legal awareness.^27^ This also highlights the importance of structured education and training to ensure that positive attitudes are supported by accurate understanding, which is essential for safe and effective implementation. However, the study was not designed to explore the degree of existing training or to evaluate targeted educational interventions; future research could investigate factors influencing awareness and the effectiveness of tailored MHL training for specific professional subgroups.

They also felt that the law would allow patients and caregivers a greater say in their healthcare. In parallel to similar research from other countries with established MHL, HCWs working in mental health showed significantly more positive attitudes to the MHL than the HCWs outside MHS.^15^ It has been suggested that this could be because the HCWs in MHS see the actual benefits of such law in action; we also believe the greater awareness of the law within the MHS HCWs in our study has a role to play in this result.

Age was not shown to be significantly associated with a positive attitude to the law in our study. An earlier investigation showed that older persons had higher mental health literacy.^28^ It is possible that older age may correspond to more experience with patients presenting with mental health issues even for non-MHS HCWs, and this translates to higher mental health literacy and therefore more positive attitudes.

Interestingly, neither gender nor country of origin had a statistically significant impact on opinions of Qatar’s MHL. Although gender differences have been reported in help-seeking behaviours and the presentation of mental illness, such differences have not been consistently observed in attitudes towards MHL, suggesting that views may be shaped more by shared professional roles and the healthcare environment than by gender alone.^29^ Previous research has suggested that attitudes to MHL vary across nations and that nations with established law systems have shown more positive attitudes.^15^ The finding that positive attitudes were cutting across country-of-origin boundaries may therefore suggest that work on mental health stigma and awareness in general has been beneficial in Qatar.^30^

Whilst more than 75% of respondents agreed that the implementation of MHL will increase patient rights and protection, lower the risk of self-harm and other harm, improve service accessibility, lessen stigma, provide a chance for an impartial second opinion, and improve life quality, more than half of participants were worried about mandatory treatment and the possibility of coercive treatment. Qatar’s MHL has, in fact, a dedicated section that legislates penalties for improper deprivation of liberties of patients with mental health disorders.^1^ The issue of coercion is a recurring theme around admissions to mental health hospitals across the world.^31–37^ In the context of poor patient insight and huge variation in international laws for protection of individual rights, this is not surprising.^38^ The fact that the majority of the respondents expressed faith in the law despite this background of general concern shows a degree of faith in health law in Qatar.

The authors further surmise that rather than being explicitly tied to law, this could be a result of the highlighted lack of awareness about the actual provisions of the MHL. Additionally, taken along with the fact that there exists law on penalties against coercive treatment within the body of the MHL reflects a more cultural anxiety about coercive laws that the MENA region has traditionally struggled with.^39^ The authors believe that informed training and awareness campaigns would address this issue. Systematic reviews of practices, systems and policies that reduce coercive practices in mental health highlight areas of good practice that we believe the MHL and health system in Qatar are based upon.^35,40^

The finding of HCWs using this survey to highlight their own stressors was an unexpected finding which the authors see as a reflection of the HCWs in the research process and its facilitation of talking about mental health. This is important in the wider context of ongoing mental health service development in Qatar, where structured national initiatives have demonstrated the value of accessible pathways for recognising and responding to mental distress.^41^ This finding, along with other findings and recommendations, will be presented to the healthcare leaders for informed actions. Continued engagement with HCWs, as reflected in this survey, will be vital for successful implementation and operationalisation of the MHL.

This study examined associations between attitudes to MHL and a limited set of demographic variables. To minimise respondent burden and maximise participation, the questionnaire was intentionally kept brief, which precluded inclusion of other potentially relevant factors such as clinical specialty, years of professional experience, exposure to psychiatric practice, and prior training in MHL. These factors may influence attitudes and could partially confound observed associations. Future research should incorporate a broader range of professional and experiential variables, which would allow more comprehensive and adjusted analyses.

The qualitative findings were not intended to explain quantitative associations in a causal sense but to provide complementary insight into how HCWs understand and experience MHL. However, integration of quantitative and qualitative findings demonstrated agreement regarding overall positive attitudes with protection of patient rights, improved quality of care, improved access to care, and reducing stigma, with a qualitative positive theme around conformity to international human rights standards, protection of rights and access to care. Similarly, low levels of self-reported training and preparedness corresponded with a theme emphasising the need for enhanced education and awareness.

This study was not grounded in a formal model of attitude formation. However, the more favourable attitudes observed among professionals with psychiatric experience may be understood in terms of increased familiarity, perceived competence, and reduced uncertainty, constructs commonly described within frameworks, such as the Health Belief Model and the Theory of Planned Behavior.^42,43^ Future research should consider applying these frameworks to enable exploration of factors that may explain attitudes and facilitate international comparisons.

## 5. STRENGTHS AND LIMITATIONS

This is the first study of this kind in this region, and the data collection tool used is based upon available evidence and has shown very good internal consistency. A sizeable sample size and free text narratives supporting Likert-scale responses are some of the strengths of the current study. Some of the limitations of the present study are that a cross-sectional design could not reliably reflect the association between a cause-and-effect relationships; and reliance on online data collection might exclude individuals with low computer proficiency and lead to bias in the data collection process.

The questionnaire was not formally validated, and no factor analysis was conducted; as a result, the total attitude score should be interpreted as a descriptive summary rather than a psychometrically validated measure of attitudinal constructs. Furthermore, respondents may have differed systematically from non-participants in terms of motivation or access to technology, which could have influenced the findings. Future studies could address this potential selection bias by using stratified random sampling and, where feasible, including multiple centres to enhance representativeness.

Several additional limitations should be acknowledged. The attitude measure used in this study was developed for the purposes of this survey and was not formally validated, which may limit its accuracy and reliability. We conducted only simple statistical comparisons, and the possibility of residual confounding remains due to unmeasured factors such as professional experience, clinical specialty, or prior MHL training. In addition, responses may have been influenced by social desirability bias, particularly given the assessment of attitudes toward government MHL by healthcare professionals employed in government-funded positions.

## 6. CONCLUSION

This study provides the first large-scale assessment of HCWs’ awareness and attitudes towards the MHL prior to its full implementation. Despite limited awareness of its detailed provisions, attitudes were consistently positive across a diverse workforce, suggesting that perceptions may be shaped more by the law’s underlying principles than by detailed operational knowledge. However, the gap between positive attitudes and limited awareness, alongside concerns regarding coercion and preparedness, underscores the need for structured training and ongoing stakeholder engagement. As implementation progresses, aligning these positive perceptions with practical understanding will be essential to ensure safe, effective, and rights-based application of the law. Future research should evaluate changes in awareness and attitudes following implementation and assess the impact of training initiatives on clinical practice.

## ACKNOWLEDGMENT

The authors thank the healthcare workers at Hamad Medical Corporation who participated in the survey and shared their perspectives on the implementation of Qatar’s Mental Health Law.

## AUTHORS’ CONTRIBUTIONS

MA and SR conceived the project. YI, SR, MA, SA and DH designed the project, applied for approvals and collected data. MO and SR carried out the analyses of the data. MA and SR wrote the initial draft. DH, YI, SA and MO reviewed the initial draft. SR, YI and MA approved and edited the final manuscript. All authors approved the final manuscript.

## CONFLICT OF INTEREST

The authors declare that they do not have any competing interests to declare in this project.

## DISCLOSURE OF AI USE

No Artificial Intelligence was used and authors take full responsibility for the final content of the manuscript.

## DATA AVAILABILITY

The authors are happy to share relevant data upon receiving a reasonable request, subject to further approval of the Institutional Review Board.

## FUNDING

No public or private funding was received for this study at any point.

## ETHICAL APPROVAL

The ethical approval for the study was sought and received from the HMC’s Institutional Review Board (approval number MRC-01-21-304).

## Figures and Tables

**Table 1.** Baseline demographic results of the participating healthcare workers and the unadjusted effect on their attitude towards mental health law (MHL) in Qatar.

Factor	Count (*n*)	%	Attitude Score		
			Mean	Test	*p*
**Age**					
18-24	5	0.5%	49.0	*F* = 1.294	0.264
25-34	393	39.1%	57.7		
35-44	384	38.2%	57.3		
45-54	166	16.5%	58.0		
55-64	54	5.4%	58.1		
65 and above	4	0.4%	63.3		
**Gender**					
Male	344	34.2%	57.9	*t* = 0.827	0.4085
Female	662	65.8%	57.4		
**Nationality**					
Qatari	23	2.3%	57.8	*t* = 0.093	0.9266
Non-Qatari	983	97.7%	57.6		
**Member of mental health services**					
Yes	217	21.6%	60.4	*t* = 0.827	< 0.0001
No	781	77.6%	56.8		

**Table 2.** Attitudes towards Qatar’s MHL among the participating individuals.

Agreement level		Agree strongly	Agree	Undecided	Disagree	Disagree strongly
Q1	I have adequate information about Qatar’s MHL.	54	301	214	308	118
		5.4%	29.9%	21.3%	30.6%	11.7%
Q2	Qatar’s MHL will protect the rights of patients admitted to hospital.	199	565	193	21	12
		19.8%	56.2%	19.2%	2.1%	1.2%
Q3	Qatar’s MHL will reduce the risk of self-harm in patients with mental illnesses.	221	567	150	30	15
		22.0%	56.4%	14.9%	3.0%	1.5%
Q4	Qatar’s MHL will reduce the risk to others from patients with severe mental health problems.	181	570	40	30	6
		18.0%	56.7%	4.0%	3.0%	0.6%
Q5	Qatar’s MHL will improve the quality of healthcare offered to patients with mental illness.	241	582	139	20	11
		24.0%	57.9%	13.8%	2.0%	1.1%
Q6	Qatar’s MHL implementation will lead to timely access to effective treatments for mental illnesses.	220	580	160	22	10
		21.9%	57.7%	15.9%	2.2%	1.0%
Q7	Qatar’s MHL implementation will ensure timely access to see a mental health professional (mental health care provider).	197	569	195	23	8
		19.6%	56.6%	19.4%	2.3%	0.8%
Q8	Qatar’s MHL will reduce stigma towards mental disorders.	203	509	207	52	18
		20.2%	50.6%	20.6%	5.2%	1.8%
Q9	Should Qatar’s MHL ensure that patients can get an independent opinion from another mental health professional if they disagree with their treating team?	183	582	194	23	10
		18.2%	57.9%	19.3%	2.3%	1.0%
Q10	Should Qatar’s MHL permit a compulsorily detained patient to appeal against their detention?	130	427	345	68	17
		12.9%	42.4%	34.3%	6.8%	1.7%
Q11	Should Qatar’s MHL permit the guardian of a compulsorily detained patient to appeal against their detention?	114	510	323	33	13
		11.3%	50.7%	32.1%	3.3%	1.3%
Q12	Should a relative or guardian be able to request an assessment for admission to a hospital under Qatar’s MHL, for a family member they are concerned about?	170	619	169	23	10
		16.9%	61.5%	16.8%	2.3%	1.0%
Q13	Should a patient, admitted to a hospital under Qatar’s MHL, be able to be given treatment against their will by the psychiatric team?	79	393	318	147	49
		7.9%	39.1%	31.6%	14.6%	4.9%
Confidence		Fully	Fairly	Somewhat	Slightly	Not at all
Q14	How confident do you feel that you have adequate training to implement Qatar’s MHL into practice?	112	244	250	176	210
		11.1%	24.3%	24.9%	17.5%	20.9%
Q15	How confident do you feel that there are currently adequate resources to implement Qatar’s MHL into practice?	132	272	249	179	153
		13.1%	27.0%	24.8%	17.8%	15.2%
Impact		A lot	Moderate	Some	Small	None
Q16	Do you believe that Qatar’s MHL may have an impact on how you assess and manage patients?	308	333	262	49	38
		30.6%	33.1%	26.0%	4.9%	3.8%

**Table 3.** Adjusted effect for background factors on attitude score towards MHL in Qatar.

Factor	β	95% Confidence Interval	*P* value
Nationality of established MHL country (Qatari vs Immigrant HCWs)	0.5669	−3.5442–4.6780	0.7870
MHS Affiliation	3.6038	2.1444 to 5.0632	< 0.00001***

*** = *p* < 0.001.
